# Genetic convergence of industrial melanism in three geometrid moths

**DOI:** 10.1098/rsbl.2019.0582

**Published:** 2019-10-16

**Authors:** Arjen E. van't Hof, Louise A. Reynolds, Carl J. Yung, Laurence M. Cook, Ilik J. Saccheri

**Affiliations:** 1 Institute of Integrative Biology, University of Liverpool, Liverpool L69 7ZB, UK; 2 Liverpool School of Tropical Medicine, Liverpool L3 5QA, UK; 3 Department of Entomology, The Manchester Museum, University of Manchester, Manchester M13 9PT, UK

**Keywords:** melanism, Lepidoptera, parallel evolution, *cortex*

## Abstract

The rise of dark (melanic) forms of many species of moth in heavily coal-polluted areas of nineteenth- and twentieth-century Britain, and their post-1970s fall, point to a common selective pressure (camouflage against bird predators) acting at the community level. The extent to which this convergent phenotypic response relied on similar genetic and developmental mechanisms is unknown. We examine this problem by testing the hypothesis that the locus controlling melanism in *Phigalia pilosaria* and *Odontopera bidentata*, two species of geometrid moth that showed strong associations between melanism and coal pollution, is the same as that controlling melanism in *Biston betularia*, previously identified as the gene *cortex*. Comparative linkage mapping using family material supports the hypothesis for both species, indicating a deeply conserved developmental mechanism for melanism involving *cortex*. However, in contrast to the strong selective sweep signature seen in British *B. betularia*, no significant association was detected between *cortex*-region markers and melanic morphs in wild-caught samples of *P. pilosaria* and *O. bidentata*, implying much older, or diverse, origins of melanic morph alleles in these latter species.

## 1. Introduction

Convergent evolution, whereby different species independently evolve similar phenotypes under similar selection pressures, can be observed throughout nature. This raises the question of whether there is also a convergence of the molecular mechanisms that control these phenotypes (parallelism; [1]). Stern [2] has argued that the repeated use of a small subset of loci, out of the total that could potentially influence any given trait, is driven by the ability of these *genetic hotspots* [3] to maximize changes to the target trait while minimizing negative pleiotropic effects. However, experimental evolution studies have also shown the opposite pattern: a diverse genetic architecture of adaptation among populations to the same environmental pressure [4]. The question of parallelism intersects with the relative importance of *de novo* mutation versus standing genetic variation, and the genetic and environmental factors that favour these sources of variation [5].

Melanism in Lepidoptera provides ample opportunity to study this problem, being widely distributed across the phylogeny, performing a range of functions, including camouflage, thermo-regulation, aposematism, sexual attraction and immunity [6]. Intriguingly, variation within the same gene (*cortex*) controls melanism for crypsis in the peppered moth, *Biston betularia* [7], and mimic wing patterns in *Heliconius* butterflies [8]. This genetic convergence suggests that *cortex*, and possibly the region surrounding it, is a genetic hotspot for lepidopteran wing pattern evolution.

*Biston betularia* is the exemplar of industrial melanism [9,10]—the rise of melanic forms associated with coal soot pollution—but the phenomenon was evident from many other moth species [11]. Two species from the same family (Geometridae) received the most attention: the scalloped hazel *Odontopera bidentata* [12–15] and the pale brindled beauty *Phigalia pilosaria* [16,17]*.* All three species have polyphagous larvae but have distinct dispersal and resting behaviour. *O. bidentata* is the least mobile, often with high local densities and the melanic form strongly associated with urbanization, *B. betularia* the most mobile and the least dense, with a broad spread of melanics across regions, and *P. pilosaria* lies between [18].

**Figure 1.**
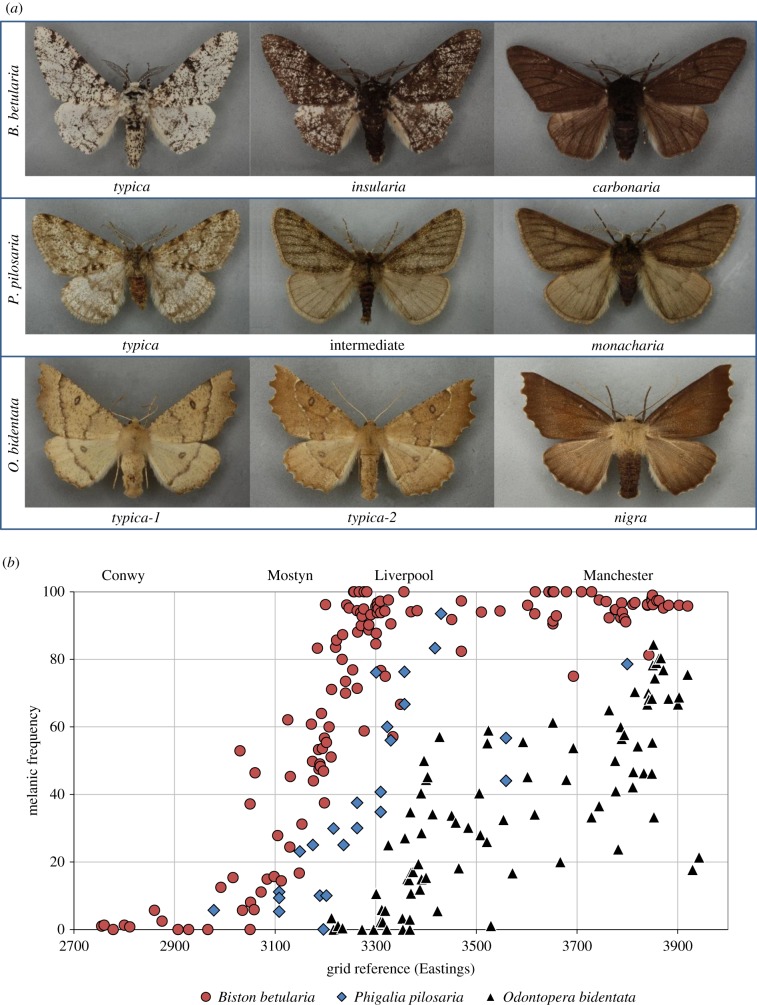
(*a*) Melanic polymorphism in *Biston betularia*, *Phigalia pilosaria* and *Odontopera bidentata*. Lighter and darker forms of *insularia* than the one shown exist. For *O. bidentata*, *typica-1* is more common in southern England, whereas *typica-2* prevails in the northwest region. The different species are scaled to similar size. Photographs from Natural History Museum Data Portal [19]. (*b*) Frequency of melanics in three moth species on a cline from west N. Wales to Cheshire and Lancashire, east of Manchester, during the period of high melanic frequency (late 1960s–mid 1970s). For *P. pilosaria*, the totals in both melanic classes (*monacharia* and intermediate) are shown. Map of transect in [20]. (Data from [12,14,21–23]).

In Britain, melanism in each of these species showed a positive association with urbanization and coal pollution, most evident during the 1960s and 1970s (figure 1). Different ecologies produce varying intensities of selection and spatial mixing, but differential visibility of the melanic versus non-melanic forms to bird predators is considered to be the driving interaction maintaining the clines in all three species [24,25]. The fully black *carbonaria* form of *B. betularia* was first reported in Manchester in 1848 and has been genetically dated to a mutation arising 30 years earlier [7]. The frequency of *carbonaria* rose rapidly towards fixation in urban centres, such as Manchester and West Yorkshire, forming a frequency cline into rural areas where the lighter *typica* form continued to predominate. At the height of coal pollution, the average frequency of the *P. pilosaria* f. *monacharia* and intermediate forms averaged over 50% in industrial England, reaching 80% in Greater Manchester and Liverpool [16,21]. Similarly, the frequency of *O. bidentata* f. *nigra* peaked at a frequency of 70–80% in Manchester, Leeds and York [12,26,27]. A complete set of melanic morph frequency records in Britain has been compiled by Cook [28].

We hypothesized that the convergent phenotypic evolution of melanic morphs in *B. betularia, P. pilosaria* and *O. bidentata*, which diverged from each other 30–45 Ma, results from parallel changes at the level of the genotype. Previous studies have shown that the traits are controlled by single loci. In *P. pilosaria*, f. *monacharia* is dominant over the lighter intermediate and typical morphs [17]. Likewise, the *nigra* form of *O. bidentata* is dominant over the lighter typical form [29]. However, the genomic regions underlying the traits had not been characterized. We do this by genetically mapping the locus that controls melanism in *P. pilosaria* and *O. bidentata*. We also assessed the degree of genotype–morph association across the identified chromosomal regions in wild samples to infer the approximate age of the melanic alleles.

## 2. Material and methods

### (a) Linkage mapping

For both species (*P. pilosaria* and *O. bidentata*), mapping families were created by crossing virgin typical females to wild-caught melanic males (table 1), equivalent to a backcross design. Larvae were reared on sallow (*Salix caprea*) and privet (*Ligustrum ovalifolium*) for *P. pilosaria* and *O. bidentata*, respectively. Both species are obligately univoltine, diapausing as pupae, with adults of *P. pilosaria* emerging from January to March, and those of *O. bidentata* in May–June.

**Table 1.** Morph and geographical origin of mapping family parents, and morph segregation in offspring. Mothers were homozygous for typical allele (t/t); fathers were typical/melanic heterozygotes (t/m).

species	mother (origin of grandmother)	father (*melanic morph*)	no. offspring
*Phigalia pilosaria*	*typica* (t/t) Guisborough, North York Moors (February 2011)	*monacharia* (t/m) Cuerden Valley Park, Preston (February 2012)	*typica*: 15 *monacharia*: 17 total: 32
*Odontopera bidentata*	*typica* (t/t) Malpas, Cheshire (May 2010)	*nigra* (t/m) Heaton Moor, Stockport (May 2011)	*typica*: 39 *nigra*: 35 total: 74

Shared anchoring markers were developed based on orthologous genes. Gene sequences and polymorphisms were obtained using consensus degenerate primers (electronic supplementary material, table S1) and primers based on low coverage Illumina HiSeq whole genome sequence (NCBI PRJNA566081 and PRJNA566083), followed by PCR-Sanger sequencing of the mapping parents. *Cortex* exon 1, which is too small and divergent to identify from sequence reads, was identified by 5′RACE (procedures as in [7]). Offspring were genotyped by Sanger sequencing or PCR-RFLP (electronic supplementary material, table S1). Linkage mapping followed [30].

### (b) Linkage disequilibrium

A previous study on *B. betularia* [31] genotyped 64 individuals, mostly from northwest England in 2002, at six linked loci (*a–f*) spread across a 1.4 Mb region, roughly centred on the causal locus, subsequently identified as *cortex* (located between *c* and *d*). For this study, we identified polymorphisms in the *P. pilosaria* and *O. bidentata* populations at five loci (*a–e*; *f* was excluded) orthologous to the original *B. betularia* markers. A sixth locus, either in one of the first exons of *cortex* (*cortE1*), or near to it, was added for all three species. Primers and reference sequences are given in electronic supplementary material, table S1. Genotypes were determined by Sanger sequencing PCR products. To achieve higher genetic resolution, linked SNPs within the same PCR fragment were used to define mini haplotypes (electronic supplementary material, table S2).

Population samples of both species (*P. pilosaria n* = 55 males; *O. bidentata n* = 95 males and females) were collected predominantly in 2011–2013, using mercury vapour and actinic light traps at different locations in northern England and Scotland. Twenty-seven *O. bidentata* from two sites were collected in 2004 using Rothampsted (tungsten bulb) light traps (electronic supplementary material, table S3 and figure S1). Morphs were classified into three categories for *P. pilosaria* (*typica*, intermediate and *monacharia*), and two categories (*typica* and *nigra*) for *O. bidentata*. DNA was extracted from individual heads (or legs for Rothampsted samples) using a phenol-chloroform method [32].

The samples come from a diversity of locations and years, such that explicit measures of linkage disequilibrium among loci are not warranted. They are, however, reasonably well mixed with respect to morph. We tested the association between alleles at each of the marker loci and melanism using an exact G-test of genic differentiation among morphs, as implemented in Genepop [33].

## 3. Results

**Figure 2.**
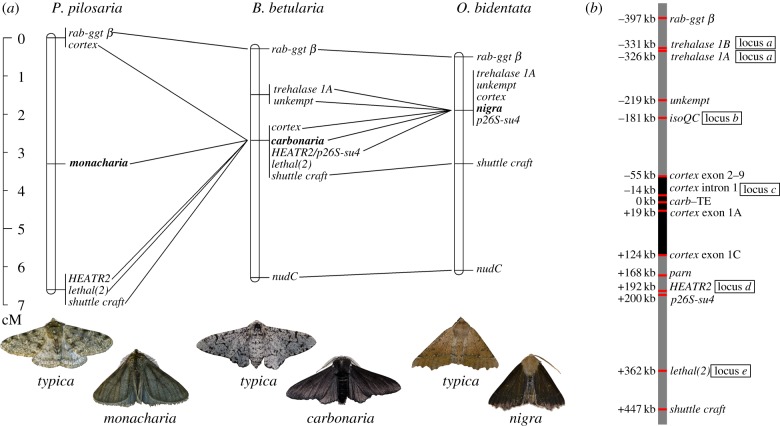
(*a*) Genomic positions, on a centimorgan scale, of the loci controlling typical–melanic polymorphism in *P. pilosaria* (*monacharia*), *B. betularia* (*carbonaria*) and *O. bidentata* (*nigra*), relative to gene orthologues (connected by lines). (*b*) Relevant genic content (11 of 56 genes) within this chromosomal region, and the position of the six genetic markers (loci *a*–*e*, and *cortex exon 1*) used to test genotype–phenotype associations in wild samples. Distances are based on *B. betularia* and are defined relative to the polymorphism controlling *typica-carbonaria* (*carb*-TE). The furthest extent of the *B. betularia cortex* region (shaded black) is extended by multiple alternative first exons (only exons 1A and 1C are shown). Locus *a* coincides with Trehalase 1A for *P. pilosaria* and *O. bidentata*, but Trehalase 1B for *B. betularia.* (Photographs by Arjen van't Hof).

Comparison of the linkage maps indicates that the loci controlling melanism in *P. pilosaria* and *O. bidentata* are orthologous to the *carbonaria* locus in *B. betularia* (figure 2). The resolution of the new maps does not specifically isolate *cortex*, within which the *carbonaria-typica* molecular polymorphism is known to reside, but the *monacharia* and *nigra* polymorphisms are both very close to *cortex*. 5′RACE produced alternative *cortex* first exons; one similar to *B. betularia cortex* exon 1A in *O. bidentata*, and a different one in *P. pilosaria*, similar to a sequence between *cortex* exon 2 and locus *c* in *B. betularia* [7]. Based on the position of this *P. pilosaria cortE1*, *monacharia* appears to map outside of *cortex* (figure 2), but the adult RNA transcript used to define this position may not be relevant for wing pattern development. In *B. betularia*, alternative first exons, associated with tissue-specific transcripts [7], greatly expand the chromosomal interval occupied by *cortex* (figure 2*b*). It is entirely plausible that the *monacharia* polymorphism falls within the sequence domain of *cortex* but is sufficiently removed from our *cortE1* marker that we were able to detect one recombination event between these positions (1/32). Conversely, the likelihood that *monacharia* lies within another gene is very small, as we also detected a recombinant between morph and *HEATR2*, and the only gene between *cortex* and *HEATR2* (*parn*) is much closer to *HEATR2* than *cortE1* (segregation patterns in electronic supplementary material, table S4).

The linkage maps are restricted to the *rab-ggt β – nudC* region because the chromosome-wide comparison is hampered by the exceptionally small and numerous (*n* = 112) chromosomes in *P. pilosaria* [34] causing markers to map outside the *rab-ggt β* – *shuttle craft* region to other linkage groups (LA Reynolds 2015, unpublished results). The large mapping intervals in *P. pilosaria* are caused by the relatively small family size resulting in 3.125 cM per recombination.

In the wild population samples, the strength of association between *cortex*-region haplotypes (as defined by genetic markers *a*–*e*) and melanism differs markedly for *B. betularia*, on the one hand, and *P. pilosaria* and *O. bidentata* on the other (table 2). As expected from previous work [7], all markers, with the exception of *e*, show a strong haplotype–morph association in *B. betularia*, but we found no significant association for either of the other two species. In *P. pilosaria*, *cortE1* alleles were significantly associated with morph when the intermediate morph was excluded from the sample (genotypes and contingency tables in electronic supplementary material, table S2). The contrast between mapping families and wild samples likely reflects narrow regions of association and a lack of extended linkage disequilibrium around melanic alleles in *P. pilosaria* and *O. bidentata*.

**Table 2.** Significance of association between marker polymorphisms and morph in wild-caught samples of three species of moth. Sample sizes: typical/melanic or typical/intermediate/melanic. *A_n_/H_e_*: number of marker alleles/expected heterozygosity. mvt: melanic versus typica only (excluding intermediate morph).

		marker locus					
species		*a*	*b*	*c*	*cortE1*	*d*	*e*
*B. betularia*	*P*-value	*	***	**	***	**	n.s.
	sample *n* (t/m)	31/31	32/32	31/28	32/32	32/29	32/31
	*A_n_/H_e_*	2/0.38	2/0.44	2/0.43	4/0.58	2/0.49	2/0.43
*A. pilosaria*	*P*-value	n.s.	n.s.	n.s.	n.s.^*mvt^	n.s.	n.s.
	sample *n* (t/i/m)	31/12/12	27/12/12	29/12/12	31/12/12	31/12/12	31/12/12
	*A_n_/H_e_*	3/0.53	2/0.36	2/0.50	4/0.65	7/0.55	8/0.69
*O. bidentata*	*P*-value	n.s.	n.s.	n.s.	n.s.	n.s.	n.s.
	sample *n* (t/m)	73/22	73/22	73/22	72/22	73/22	73/22
	*A_n_/H_e_*	2/0.20	2/0.47	2/0.42	6/0.66	3/0.64	5/0.76

Significance levels: n.s., not significant, *≤0.05, **≤0.01, ***≤0.001.

## 4. Discussion

Our results imply the conservation of a developmental master switch for melanism in geometrid moths spanning 30–45 Myr, the estimated age of the subfamily Ennominae [35]. Interestingly, melanic forms in several other ennomine moths are inherited as single locus dominants [25]. This functional conservation in generating wing pattern polymorphisms extends to butterflies [8], but the data are currently too sparse to evaluate the general role of *cortex* for melanism across phylogenetic space [36]. Compared to the genetics of melanism in *Drosophila*, where pigmentation differences within and between species have been traced to *cis*-regulators of a subset of pigment synthesis genes [37], the emerging pattern for *cortex* suggests greater developmental constraints in the Lepidoptera. This may relate to the greater complexity of the lepidopteran wing surface, in which the colour and structure of scales are intricately linked [38]. As a high-level cell-cycle regulator, which appears to determine pattern boundaries rather than pigment *per se*, tinkering with *cortex* expression may avoid deleterious pleiotropic effects of mutations to melanin pathway genes downstream. A tendency for *cortex* mutations to produce dominant melanism, through a positive association with upregulation [7], would also be an advantage.

The origin of industrial melanic forms is relevant to understanding the factors maintaining these polymorphisms. Kettlewell [11] identified over 100 British moth species in which melanic forms had increased in frequency and made an attempt to classify these into forms that pre-date the industrial revolution and those that were recorded during this period, implying a recent mutational origin. The difficulty is that absence of a pre-1800s record is not, in general, strong evidence for the complete absence of the form. *Biston betularia* f. *carbonaria* stands out as one of the forms convincingly not observed prior to the 1840s [39]. The historical record for *P. pilosaria* f. *monacharia* and *O. bidentata* f. *nigra* is less definitive. The occurrence of *monacharia* (and ‘intermediate’) at appreciable frequencies in rural settings suggests that they both have a non-industrial origin. However, Kettlewell [11] classifies *nigra* as an industrial melanic from London and the north of England.

The absence of a strong association between melanic forms and haplotype in wild-caught samples of *P. pilosaria* and *O. bidentata* implies that neither *monacharia* or *nigra* are owing to a singular mutation event occurring within the past 200–300 years (generations). We have not identified the precise locations of the functional sequence polymorphisms, but the linkage maps indicate that they are contained within the approximately 700 kb region surveyed, and therefore, less than 200 kb from one of the markers (*a–e*). This resolution should detect linkage disequilibrium generated by single mutation events within this time period, as demonstrated by the *B. betularia typica*-*carbonaria* polymorphism, dated to the early 1800s [7]. Based on this evidence, *monacharia* and *nigra* are likely to be much older alleles, recombined onto a diversity of haplotype backgrounds, before spreading with coal pollution. An alternative explanation for the lack of a strong genotype–phenotype association in our samples is that *monacharia* and *nigra* are relatively young but each caused by more than one mutation [5].

Intermediate melanic morphs (*insularia* complex) of *B. betularia*, which are controlled by alleles at the *carbonaria* locus [40], were not included in this study, but we have previously detected an absence of association between *cortex-*region haplotype and morph in a small wild-collected sample of the darkest *insularia* [7]. A temporary peak in *insularia* during the early *carbonaria* increase suggests *insularia* phenotypes were present when *carbonaria* first arose [28]. They are therefore more like the melanics in the other two species, as distinct from the uniquely industrial character of *carbonaria*.

We have shown that parallel phenotypic clines across three co-occurring species, reflecting rapid adaptive responses to the same anthropogenic factor, relied on genetic variation at the same locus. The extent of the genetic parallelism remains to be resolved. Does the apparently much older variation in *P. pilosaria* and *O. bidentata* involve transposable element insertions, as for *B. betularia carbonaria* [7], or some other type of *cis*-regulatory element, or coding variation, influencing *cortex* functional expression?

## Supplementary Material

Primers and amplicon sequences

## Supplementary Material

Supplementary text

## Supplementary Material

Genotypes of wild-caught samples

## Supplementary Material

Mapping families

## Supplementary Material

Alternative morphs of Odontopera bidentata and Phigalia pilosaria

## Data accessibility

All the data are available as electronic supplementary material or via NCBI.
