# Supplementary material for: Genetic convergence of industrial melanism in three geometrid moths
Source: Biol Lett. 2019 Oct 16;15(10):20190582. doi: 10.1098/rsbl.2019.0582 (PMC6832188; doi:10.1098/rsbl.2019.0582)
Supplement: Supplementary text [file rsbl20190582supp2.docx]

**Genetic convergence of industrial melanism in three geometrid moths**

Arjen E van’t Hof, Louise A Reynolds, Carl J Yung, Laurence M Cook, Ilik J Saccheri

**Supplementary figures and tables**


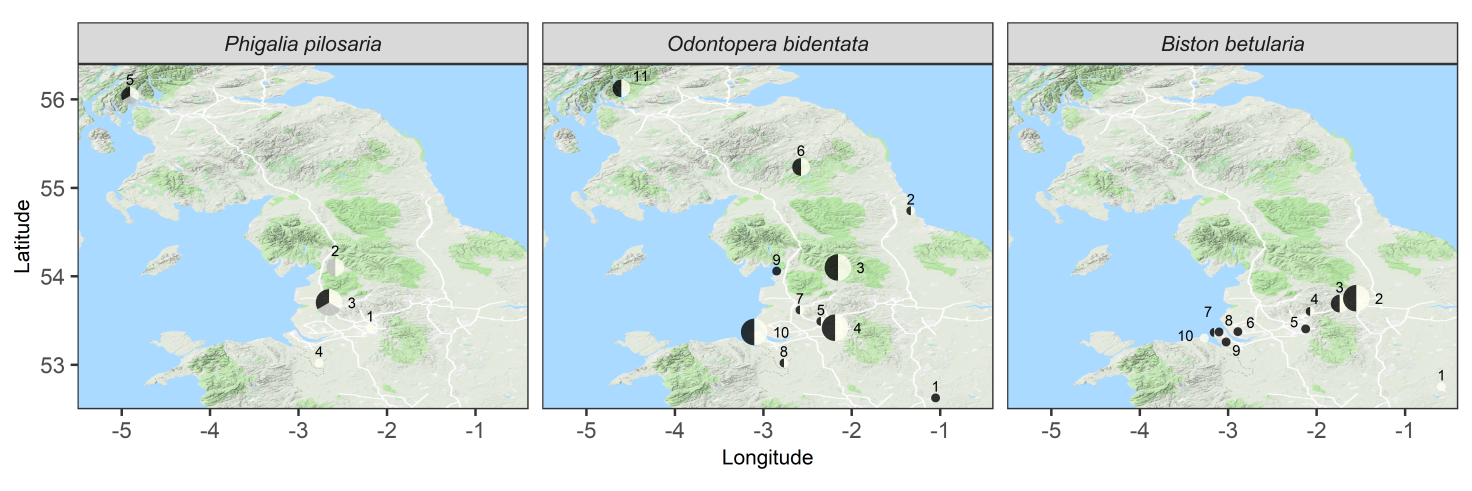


**Figure S1.** Maps of northern Britain showing the location of sampling sites for each species. The size of the circles is proportional to the sample size, and the colour indicates whether the sample contained typical (cream), intermediate (grey), and/or melanic (black) morphs. Numbers correspond to location # in Table S3.

**Table S3.** Summary of wild-caught samples used in this study.

| **Loc #** | **Place Collected** | **Long (W)** | **Lat (N)** | **n** | **typical** | **melanic** | **inter** |
| --- | --- | --- | --- | --- | --- | --- | --- |
| ***Phigalia pilosaria*** | |  |  |  |  |  |  |
| 1 | Heaton Moor, Stockport | 2°11'44” | 53°25'17” | 2 | 2 | 0 | 0 |
| 2 | Millhouses, Lancashire | 2°35'10” | 54°06'14” | 8 | 5 | 0 | 3 |
| 3 | Cuerden Valley Park, Lancashire | 2°39'37” | 53°42'28” | 33 | 15 | 11 | 7 |
| 4 | Malpas, Cheshire | 2°46'07” | 53°01'24” | 1 | 1 | 0 | 0 |
| 5 | Ardentinny, Argyll | 4°54'38” | 56°02'39” | 11 | 8 | 1 | 2 |
| ***Odontopera bidentata*** | |  |  |  |  |  |  |
| 1 | Leicester, East Midlands | 1°03'25” | 52°37'59” | 1 | 0 | 1 | na |
| 2 | Castle Eden Dene, County Durham | 1°20'05” | 54°44'45” | 2 | 1 | 1 | na |
| 3 | Malham Tarn Wood, Yorkshire Dales | 2°09'50” | 54°06'04” | 31 | 27 | 4 | na |
| 4 | Heaton Moor, Stockport | 2°11'44” | 53°25'17” | 15 | 6 | 9 | na |
| 5 | Swinton, Greater Manchester | 2°21'08” | 53°29'47” | 2 | 1 | 1 | na |
| 6 | Kielder Forest, Northumberland | 2°34'49” | 55°14'15” | 8 | 7 | 1 | na |
| 7 | Heath Charnock, Lancashire | 2°35'26” | 53°37'21” | 3 | 2 | 1 | na |
| 8 | Malpas, Cheshire | 2°46'07” | 53°01'24” | 5 | 4 | 1 | na |
| 9 | Morecambe, Lancashire | 2°51'05” | 54°03'58” | 1 | 0 | 1 | na |
| 10 | Greasby, Wirral | 3°06'29” | 53°22'28” | 19 | 18 | 1 | na |
| 11 | Rowardennan, Stirlingshire | 4°36'40” | 56°07'45” | 8 | 7 | 1 | na |
| ***Biston betularia*** | |  |  | 95 |  |  |  |
| 1 | Morkery Wood, Lincolnshire | 0°35'44” | 52°45'22” | 2 | 2 | 0 | 0 |
| 2 | Middleton Park, Leeds | 1°33'14” | 53°45'17” | 39 | 19 | 20 | 0 |
| 3 | Kirklees Hall, West Yorkshire | 1°44'43” | 53°41'52” | 9 | 5 | 4 | 0 |
| 4 | Ogden Reservoir, Rochdale | 2°04'48” | 53°36'22” | 2 | 1 | 1 | 0 |
| 5 | Woodbank Park, Stockport | 2°07'51” | 53°24'37” | 2 | 0 | 2 | 0 |
| 6 | Calderstones Park, Liverpool | 2°53'50” | 53°22'51” | 1 | 0 | 1 | 0 |
| 7 | Caldy, Wirral | 3°09'58” | 53°22'01” | 4 | 3 | 1 | 0 |
| 8 | Greasby, Wirral | 3°06'28” | 53°22'27” | 2 | 0 | 2 | 0 |
| 9 | Burton Wood, Wirral | 3°01'47” | 53°15'50” | 1 | 0 | 1 | 0 |
| 10 | Mostyn, Flintshire | 3°16'26” | 53°18'20” | 2 | 2 | 0 | 0 |

**Supplementary materials & methods**

***Biston betularia* linkage mapping**

Markers on the *B. betularia* linkage map were partly mapped and partly assigned to mapping intervals. Markers positioned between two non-recombinant markers have a segregation pattern identical to the mapped surrounding markers and therefore the same centiMorgan position. The mapping position of *Trehalase 1A* and *unkempt* could be inferred from the segregation pattern of surrounding fully-linked mapped genes (*Trehalase 1B* and *isoQCI)*, and the position of *p26s-su4* from *HEATR2* and *lethal (2)*. *Trehalase 1B* and *isoQC* are not shown in the *B. betularia* linkage map (Fig. 2a) because they were not mapped in *P. pilosaria* and *O. bidentata*.

**Linkage disequilibrium markers**

Locus *c*, situated in the large intron 1 of *cortex*, was obtained using non-coding sequence similarity because it lies too far from exon 1 and 2 to be reached by local read assembly. Loci *a, b, d, e* sequences lie near or inside exons and were obtained as described for the linkage mapping markers. *Cortex* exon 1, which is too small and divergent to identify from sequence reads, was identified by 5’RACE and extended by local read assembly.
