# Supplementary figures and images for: Genetic convergence of industrial melanism in three geometrid moths
Source: Biol Lett. 2019 Oct 16;15(10):20190582. doi: 10.1098/rsbl.2019.0582 (PMC6832188; doi:10.1098/rsbl.2019.0582)

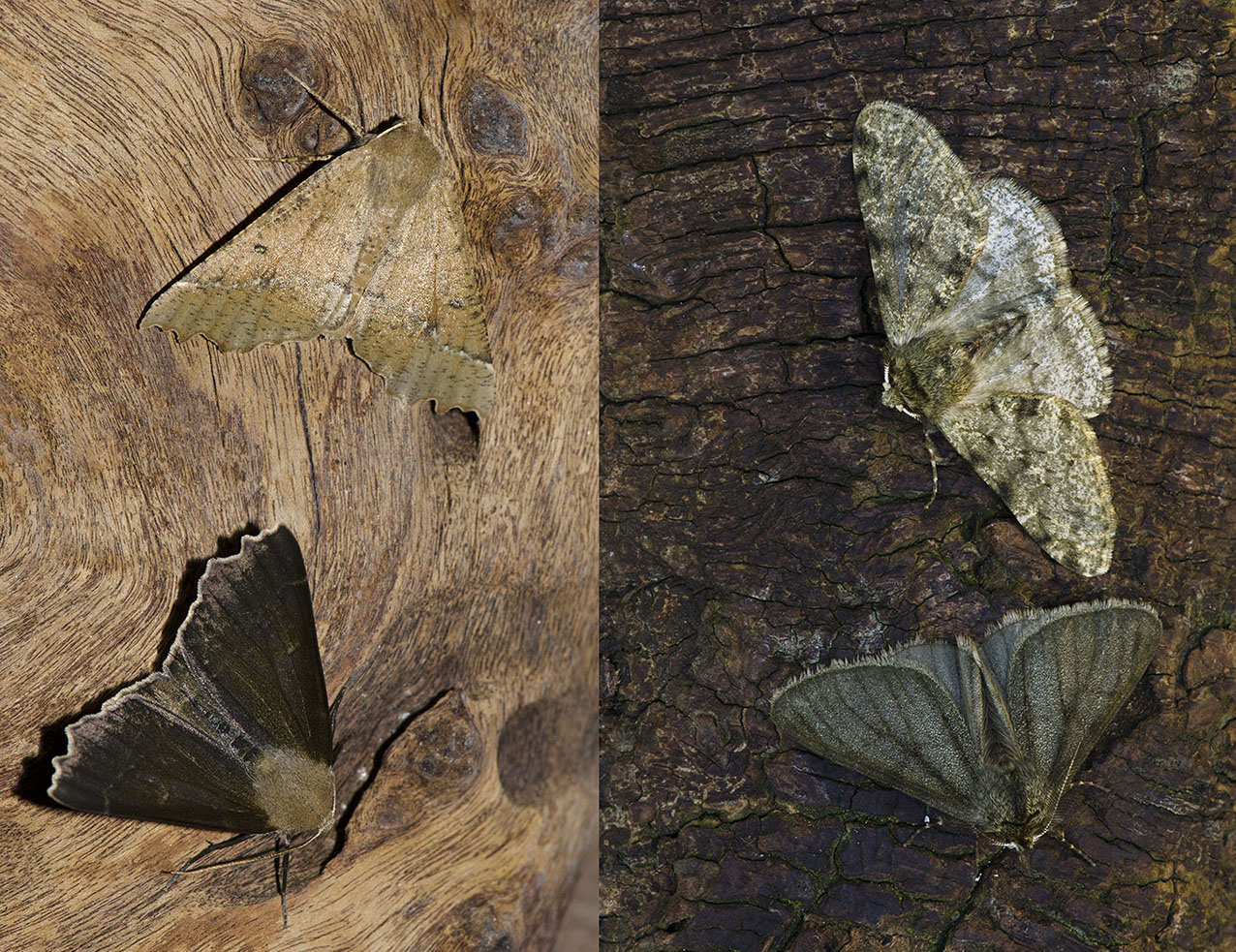

Supplement: Alternative morphs of Odontopera bidentata and Phigalia pilosaria [file rsbl20190582supp5.jpg]
